# A Rare and Complex Diagnostic Challenge: Herpes Zoster Ophthalmicus With Secondary Orbital Apex Syndrome in a 65-Year-Old Male

**DOI:** 10.7759/cureus.82219

**Published:** 2025-04-14

**Authors:** Gunjan Awatramani, Ghazal Talal Saeed, Montaser Nabeeh Al Smady, Sara Tahlak, Heba Talal Saeed, Rajesh Shah, Pramod Warhekar

**Affiliations:** 1 College of Medicine, Mohammed Bin Rashid University of Medicine and Health Sciences, Dubai, ARE; 2 Emergency Medicine, Dubai Health, Dubai, ARE; 3 Hamdan Bin Mohammed College of Dental Medicine, Dubai Health, Dubai, ARE; 4 Radiology, Mediclinic City Hospital, Dubai, ARE; 5 Ophthalmology, Mediclinic City Hospital, Dubai, ARE

**Keywords:** herpes zoster ophthalmicus, orbital apex syndrome, ptosis, retro-orbital pain, upper lid edema

## Abstract

Herpes zoster (HZ) results from the reactivation of the varicella zoster virus (VZV) in sensory ganglia, with immunosuppression and aging being major risk factors. A subtype, HZ ophthalmicus (HZO), involves the ophthalmic division of the trigeminal nerve and can lead to significant ocular complications. Orbital apex syndrome (OAS), a rare condition involving multiple cranial nerve dysfunction due to involvement of the orbital apex, may complicate HZO.

A 65-year-old male with a history of type 2 diabetes mellitus, benign prostatic hyperplasia, migraines, and bipolar disorder presented with a two-day history of a severe right retro-orbital headache, lacrimation, nausea, dizziness, and photophobia. Initial work-up suggested a cluster headache. However, he developed a vesicular rash on the right side of his forehead, along with a swollen right eyelid and red eye. On the fourth day of admission, a diagnosis of HZO and OAS was made, and antiviral therapy with intravenous acyclovir and methylprednisolone was added to his pain management regimen.

OAS is a rare but severe complication of HZO, characterized by painful ophthalmoplegia, vision loss, and cranial nerve dysfunction. If left untreated, OAS can be fatal if it involves the cavernous sinus. The diagnosis is confirmed through clinical examination and neuroimaging, which may reveal orbital myositis, optic nerve abnormalities, and perineural enhancement. OAS can be precipitated by comorbidities such as diabetes, and its onset typically occurs 10-14 days after the rash. Treatment consists of analgesics, antiviral therapy, and corticosteroids. Early recognition and aggressive management are essential to prevent long-term complications, such as postherpetic neuralgia and permanent vision loss.

## Introduction

Herpes zoster (HZ) occurs when the varicella zoster virus (VZV) reactivates from its dormant state in sensory ganglia, with immunosuppression and aging being key risk factors. A subtype of HZ, called HZ ophthalmicus (HZO), affects the ophthalmic division of the fifth cranial trigeminal nerve (V). Between 4% and 20% of HZ patients develop HZO, and around 50% of those with HZO experience ocular involvement, such as conjunctivitis, uveitis, episcleritis, keratitis, and retinitis. Up to 25% of these individuals may experience chronic or recurrent disease [1]. Orbital apex syndrome (OAS) is a condition affecting multiple cranial nerves due to involvement of the orbital apex, caused by infections, inflammation, trauma, tumors, or other factors. Symptoms include vision loss, ophthalmoplegia, and pain. Diagnosis and management require a multidisciplinary approach to identify the underlying cause [2]. In older adults, such as the 65-year-old male discussed in this case, the risk of severe sequelae from both HZO and OAS is heightened, requiring prompt recognition and aggressive management to prevent long-term morbidity. This paper examines the diagnostic and therapeutic challenges of managing a patient with concurrent HZO and OAS, highlighting the importance of early intervention in reducing the risk of permanent vision loss and other complications.

## Case presentation

A 65-year-old male patient with a known history of type 2 diabetes, benign prostatic hyperplasia, migraines, and bipolar disorder presented to the Emergency Department complaining of severe right retro-orbital pain and lacrimation of the eye for the past two days. The pain was associated with nausea, dizziness, and photophobia. He was vitally stable, and no neurological deficit was evident on examination by the neurologist. An initial diagnosis of cluster headaches was suggested, for which the patient was started on sumatriptan, oral corticosteroids, and high-flow oxygen. He was admitted for further management and observation, with unremarkable labs on presentation.

Imaging was performed, and computed tomography angiography (CTA) and computed tomography venography (CTV) were unremarkable, showing no evidence of cerebral venous sinus thrombosis (CVST). Magnetic resonance angiography (MRA) and magnetic resonance venography (MRV) were not performed on admission due to the patient’s claustrophobia. 

The patient was next reviewed by the ophthalmologist to rule out glaucoma as a cause of retro-orbital pain. No ophthalmic cause of the headache was found initially, with the only significant findings being the presence of bilateral non-proliferative diabetic retinopathy (NPDR) on wide-angle fundus photography. Anterior segment examination, intraocular pressure (IOP), and optical coherence tomography (OCT) of the optic nerve were within normal limits.

The right retro-orbital pain did not improve with pain medications, including intravenous morphine and oxygen therapy. On the following day, the patient developed vomiting and was unable to tolerate oral intake. Pregabalin was added for pain management.

He subsequently developed a red and watery right eye with a swollen eyelid. Magnetic resonance imaging (MRI) of the brain with contrast was performed under general anesthesia and revealed no intracranial abnormality. A right lateral frontal sinus retention cyst was noted on MRI, for which the ear, nose, and throat (ENT) team was consulted and ruled out as a cause of his headache. No CVST was seen on MRI.

The diagnosis was then revised to trigeminal autonomic cephalgia, specifically hemicrania continua. Indomethacin treatment was initiated, and additional issues, such as hyponatremia, were addressed by the internal medicine team. The patient reported significant improvement in pain thereafter.

Four days after admission, the ophthalmology team reexamined him and found a faint erythematous rash on the right side of the scalp and forehead. A faint rash along the V1 dermatome was noted, but no definite vesicles were present at that time. A diagnosis of HZO was made, and antiviral therapy was initiated with intravenous acyclovir 750 mg three times daily. Inflammatory markers, including C-reactive protein (CRP) and erythrocyte sedimentation rate (ESR), were performed; CRP was elevated to 130 mg/dL at the time of HZO diagnosis. The infectious diseases team was involved, and isolation precautions were advised, along with post-exposure prophylaxis with the varicella vaccine for family members.

**Figure 1:**
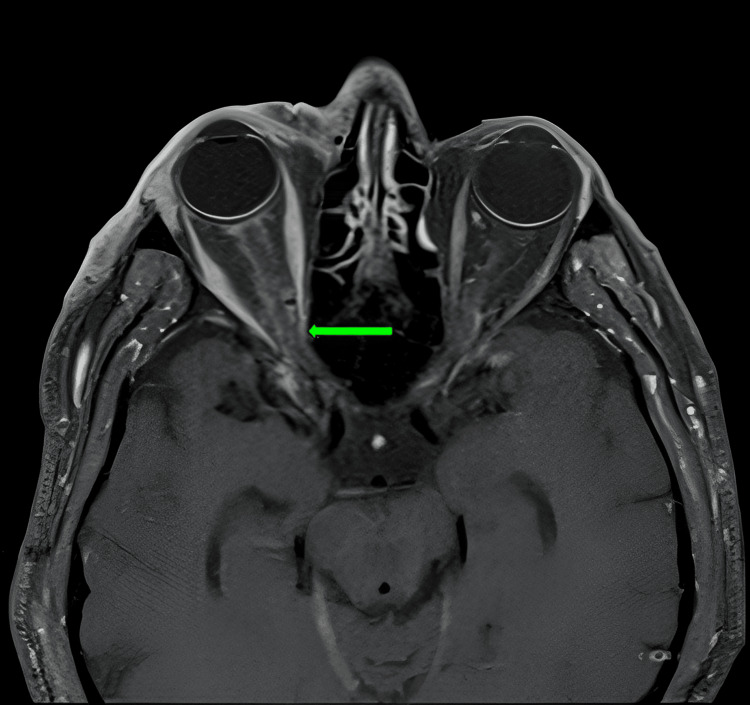
An axial MRI image with contrast demonstrating enhancement of the perineural sheath of the optic nerve in the right eye (green arrow).

**Figure 2:**
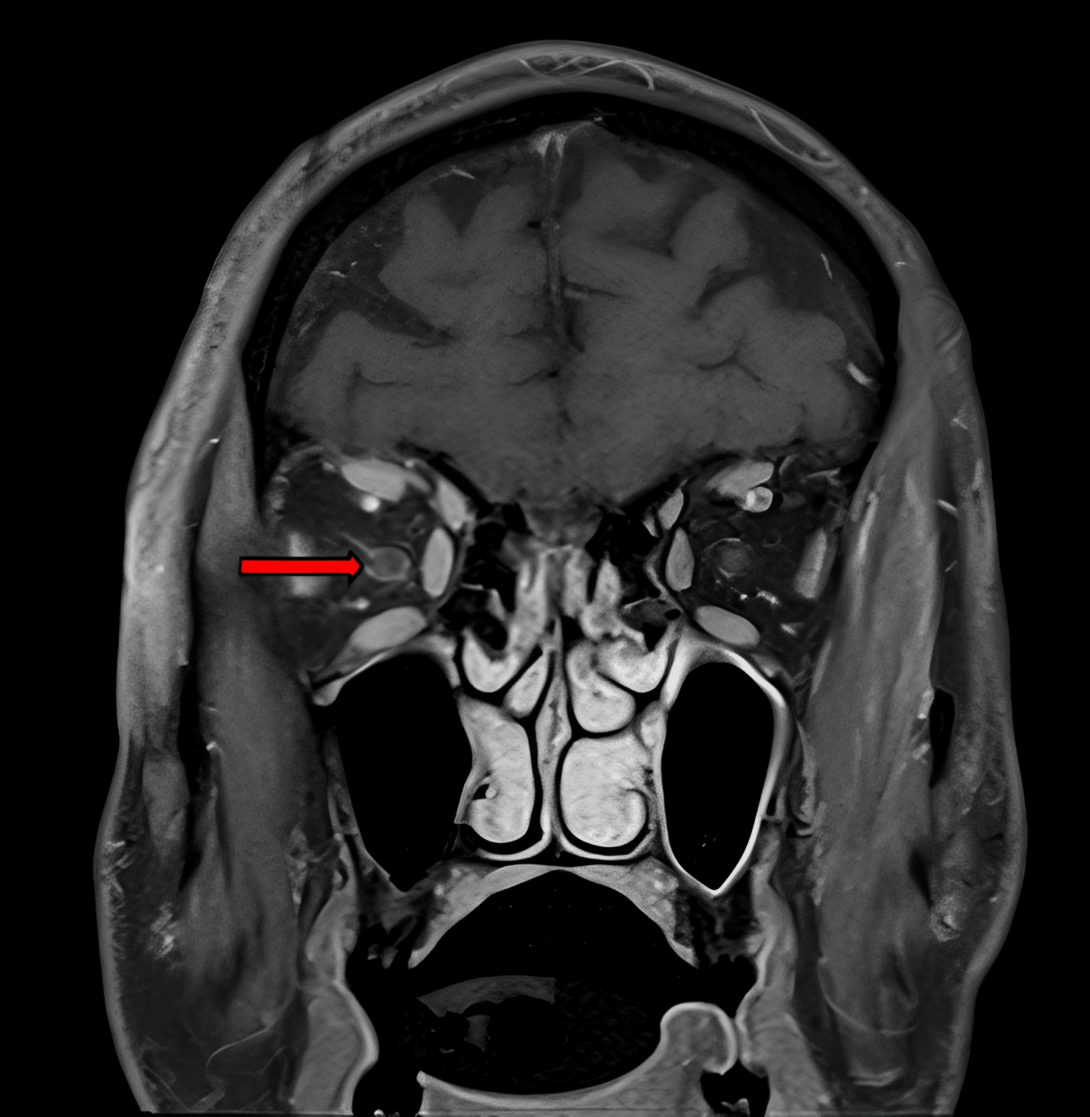
A coronal MRI image with contrast demonstrating enhancement of the perineural sheath of the optic nerve in the right eye (red arrow).

The following day, the ophthalmology team reexamined the patient. The right eye showed congestion, chemosis, bilateral quiet pseudophakia, swelling, and ptosis of the upper eyelid. A diagnosis of partial third nerve palsy was also suspected due to the presence of mild vertical diplopia for distance and mild right hypertropia after lifting the right upper eyelid. Mechanical restriction of ocular movement was noted in abduction and depression. The cornea was clear, with quiet anterior chambers, and the pupils were normal with no relative afferent pupillary defect. Visual acuity was 0.6 in the right eye and 1.0 in the left, with normal intraocular pressures bilaterally. Fundus examination and disc OCT were unremarkable bilaterally. A diagnosis of HZO with early right-sided OAS was made, and intravenous methylprednisolone was added to the existing acyclovir therapy, along with pain management. MRI with contrast showed orbital myositis and enhancement of the perineural sheath of the optic nerve in the right eye (Figures 1, 2). A right orbital preseptal collection was also present, suggestive of fluid accumulation due to compression of the orbital veins at the apex of the orbit.

The dermatologist reviewed the patient, agreed with the diagnosis, and advised continuation of the same treatment without the addition of topical medications. The vesicles were fewer in number the following day, and the rash was subsiding with no evidence of further progression to other areas of the body. The patient showed signs of improvement, with reduced pain, reduced swelling, and no double vision. 

The ophthalmology team reexamined him and noted improvement in the right upper lid edema and ptosis. The vertical diplopia had also improved, and best corrected visual acuity (BCVA) increased to 0.9 in the right eye. He was subsequently switched to oral prednisolone.

During treatment, the patient experienced multiple episodes of sharp, electric, shock-like pains along the right side of the forehead. Intravenous tramadol and pregabalin (Lyrica) were prescribed to manage the pain, which was suspected to be postherpetic neuralgia. At the time of discharge, the right upper lid edema, rash, and chemosis had completely resolved, along with the ptosis and diplopia. The patient was discharged with valacyclovir for three weeks and tapering doses of oral prednisone, starting with 60 mg for three days and gradually tapering to 5 mg over 15 days. 

He was reviewed again in the outpatient department and reported no complaints at the time. On local examination, there was dry scabbing of healed herpetic vesicles over the right temporal area. Neurological examination revealed minimal residual ptosis of the right upper eyelid with mild edema of the lower right eyelid. Facial nerve function was intact bilaterally.

## Discussion

We present a rare case of herpes zoster ophthalmicus complicated by early-onset OAS in a patient with multiple comorbidities. OAS involves multiple cranial neuropathies along with optic nerve dysfunction. The condition is diagnosed based on clinical history and physical examination findings, along with radiological confirmation [3]. 

Anatomically, the orbital apex is the posterior part of the orbit positioned at the craniofacial junction, where the four orbital walls converge. It incorporates the superior orbital fissure and the optic canal. The optic canal transmits the ophthalmic artery and the optic nerve. The superior orbital fissure, located lateral to the optic canal, is classified into superior, middle, and inferior portions. The superior portion transmits the frontal nerve (CN V1), lacrimal nerve (CN V1), recurrent meningeal artery, superior branch of the ophthalmic vein, and trochlear nerve (CN IV). The middle portion transmits the abducens nerve (CN VI), nasociliary nerve (CN V1), and the superior and inferior branches of the oculomotor nerve (CN III). The inferior portion transmits the inferior branch of the ophthalmic vein [4,5]. Clinically, OAS is symptomatically related to cavernous sinus syndrome and superior orbital fissure syndrome. 

The etiologies of OAS include inflammatory, infectious, iatrogenic, traumatic, neoplastic, and hormonal causes. Orbital signs such as proptosis, chemosis, and periorbital edema may occur, but the characteristic manifestation of OAS is painful external ophthalmoplegia with vision loss. Symptoms reported by patients include reduced or total vision loss on the affected side, inability to open the eye, facial pain, and diplopia (vertical or horizontal). These manifestations result from the involvement of the aforementioned nerves that course through the orbital apex. 

Neuroimaging, when performed, may reveal several abnormalities such as extraocular muscle enlargement, optic nerve anomalies, and perioptic nerve sheath enhancement [6]. Similarly, in our case, orbital MRI with contrast and MRV demonstrated orbital myositis with mid-enhancement of the optic nerve perineural sheath, without any evidence of cavernous sinus thrombosis. This is an important finding, as venous sinus thrombosis can occur as a complication of OAS secondary to herpes zoster ophthalmicus and is associated with a poor prognosis [7].

Several case reports have described OAS. Some of the comorbidities reported in these patients include diabetes mellitus, leukemia, and immunodeficiency syndromes, with our patient having diabetes mellitus. The common theme is a weakened immune response, allowing for the reactivation of the herpes zoster virus. It is estimated that the average time of onset between the rash and the development of OAS is approximately 10 days [4]. However, in our patient, the diagnosis of OAS was made one day after the rash appeared.

The treatment of choice is a combination of antiviral and corticosteroid therapy, which can be given through the oral or intravenous route, with an average treatment duration of 7-10 days. Initiation of treatment within 72 hours of rash onset has been shown to reduce the incidence of post-herpetic complications [8]. Our patient received two weeks of intravenous antiviral therapy during admission, followed by a three-week course of oral antiviral therapy. He was initially given intravenous methylprednisolone during admission for three days, and upon discharge, he was prescribed tapering doses of oral prednisone for three weeks.

It is imperative to consider herpes zoster ophthalmicus as a differential diagnosis in patients who present with eye pain and have a background of weakened immune response [9]. Our patient presented with severe right retro-orbital headache and lacrimation of the eye for two days, which was associated with nausea, dizziness, and photophobia. Given the acute presentation, variability of symptoms, and the history of migraines, the differentials at the time included cluster headache and atypical migraine, which were later ruled out due to failure to respond to the appropriate management. Hence, trigeminal autonomic cephalalgia was considered, and the patient showed improvement with the treatment initiated. After the onset of the rash, herpes zoster ophthalmicus was considered and confirmed as the cause of the patient’s presentation. The initial symptoms were due to the prodromal phase, which can divert physicians toward other differential diagnoses.

Permanent damage, such as vision loss, visual field defects, subnormal visual acuity, and impaired color vision, is a potential outcome for patients with OAS unless treated early in the clinical course [10,2].

## Conclusions

This case report highlights the importance of early diagnosis and treatment of HZO complicated by OAS. Multidisciplinary management involving antiviral therapy, corticosteroids, and pain control is crucial for improving outcomes and minimizing the risk of permanent damage. Early recognition of the prodromal symptoms of HZO, supplemented by appropriate imaging, is crucial in the effective management of OAS.
